# PYY is a negative regulator of bone mass and strength

**DOI:** 10.1016/j.bone.2019.07.011

**Published:** 2019-10

**Authors:** Victoria D. Leitch, Mary Jane Brassill, Sofia Rahman, Natalie C. Butterfield, Pattara Ma, John G. Logan, Alan Boyde, Holly Evans, Peter I. Croucher, Rachel L. Batterham, Graham R. Williams, J.H. Duncan Bassett

**Affiliations:** a Molecular Endocrinology Laboratory, Department of Medicine, Hammersmith Campus, Imperial College London, London W12 0NN, United Kingdom; b Centre for Obesity Research, University College London, London WC1E 6JF, United Kingdom; c Queen Mary University of London, Oral BioEngineering, Bart's and The London School of Medicine and Dentistry, London E1 4NS, United Kingdom; d Sheffield Myeloma Research Team, University of Sheffield, Sheffield S10 2RX, United Kingdom; e The Garvan Institute of Medical Research and St. Vincent's Clinical School, University of New South Wales Medicine, Sydney, New South Wales 2010, Australia; f National Institute of Health Research, University College London Hospitals Biomedical Research Centre, London Q1T 7DN, United Kingdom

**Keywords:** Bone mineral density, Osteoporosis, Fracture, PYY, Osteoblast

## Abstract

**Objective:**

Bone loss in anorexia nervosa and following bariatric surgery is associated with an elevated circulating concentration of the gastrointestinal, anorexigenic hormone, peptide YY (PYY). Selective deletion of the PYY receptor Y1R in osteoblasts or Y2R in the hypothalamus results in high bone mass, but deletion of PYY in mice has resulted in conflicting skeletal phenotypes leading to uncertainty regarding its role in the regulation of bone mass. As PYY analogs are under development for treatment of obesity, we aimed to clarify the relationship between PYY and bone mass.

**Methods:**

The skeletal phenotype of *Pyy* knockout (KO) mice was investigated during growth (postnatal day P14) and adulthood (P70 and P186) using X-ray microradiography, micro-CT, back-scattered electron scanning electron microscopy (BSE-SEM), histomorphometry and biomechanical testing.

**Results:**

Bones from juvenile and *Pyy* KO mice were longer (*P* < 0.001), with decreased bone mineral content (*P* < 0.001). Whereas, bones from adult *Pyy* KO mice had increased bone mineral content (*P* < 0.05) with increased mineralisation of both cortical (*P* < 0.001) and trabecular (*P* < 0.001) compartments. Long bones from adult *Pyy* KO mice were stronger (maximum load *P* < 0.001), with increased stiffness (*P* < 0.01) and toughness (*P* < 0.05) compared to wild-type (WT) control mice despite increased cortical vascularity and porosity (*P* < 0.001). The increased bone mass and strength in *Pyy* KO mice resulted from increases in trabecular (*P* < 0.01) and cortical bone formation (*P* < 0.05).

**Conclusions:**

These findings demonstrate that PYY acts as a negative regulator of osteoblastic bone formation, implicating increased PYY levels in the pathogenesis of bone loss during anorexia or following bariatric surgery.

## Highlights

• Juvenile *Pyy KO* mice have accelerated growth and reduced bone mass.
• Adult *Pyy KO* mice have increased bone mass and mineralisation.
• Adult *Pyy KO* mice have increased osteoblastic bone formation and mineral apposition.
• Adult *Pyy KO* mice have increased bone strength despite increased porosity.
• Elevated PYY implicated in the pathogenesis of weight loss related osteoporosis.

## Abbreviations

BFR: bone formation rate
BMC: bone mineral content
BMD, bone mineral density
BSE-SEM: back-scattered electron scanning electron microscopy
BV/TV: bone volume as a percentage of tissue volume
Ca.Dm: mean canal diameter
C.Th: cortical thickness
Ct.Po: cortical porosity
KO: knock out
Ma.Dm: marrow cavity diameter
MAR: mineral apposition rate
MS/BS: mineralising surface as a percentage of bone surface
Oc.S/BS: Osteoclast surface per bone surface
Oc.N/BS: Osteoclast number per bone surface
PECAM-1: Platelet endothelial cell adhesion molecule
PYY: peptide-YY
RBC: red blood cell
Tb.N: trabecular number
Tb.Th: trabecular thickness
TCV: trans-cortical vessel
WT: wild-type

## 1 Introduction

Bone loss, and increased fracture risk, are features of the metabolic bone disease caused by caloric restriction or poor nutrition. Decreased bone mineral density (BMD) and increased susceptibility to fracture occur in up to 90% of cases of anorexia nervosa, following bariatric surgery, and in animal models of caloric restriction [[1], [2], [3], [4], [5], [6], [7], [8]].

One potential mechanism for this deterioration in BMD is through the anti-anabolic actions of peptide YY (PYY). A member of the neuropeptide Y family, PYY is expressed in the brain, gastrointestinal tract and pancreas, with the highest tissue expression levels in the neuroendocrine L-cells of the distal gut. PYY has two active forms, the full length protein PYY(1–36), and the more abundant truncated form, PYY(3–36) that results from rapid N-terminal cleavage by the ubiquitous protease dipeptidyl peptidase-4 (DPP-4). These PYY peptides circulate in plasma and act through Y receptors, Y1, 2 and 5, to regulate food intake and beta-cell mass [[8], [9], [10], [11], [12], [13]]. Systemic PYY levels are low in the fasted state and increase in response to nutrient ingestion [10,14,15]. Paradoxically, the level of circulating post-prandial PYY is increased following bariatric surgery, particularly procedures that result in the rapid delivery of nutrients to L-cells in the ileum and colon such as Roux-en-Y gastric bypass [16]. The elevated PYY levels following bariatric surgery may contribute to reduced appetite and food intake, as exogenous administration of a PYY(3–36) analogue leads to reduced food intake in both animal models and human clinical trials [[17], [18], [19]]. Consequently, PYY analogues are currently under investigation for the treatment of obesity [20].

An inverse relationship has been reported between circulating PYY levels and BMD in patients with anorexia nervosa and following bariatric surgery [21,22]. Osteoblast-specific deletion of the Y1 receptor promoted osteoblast differentiation and mineralisation through increased *Runx2* and *Osterix* expression [23]. Consistent with this, global *Y1R*^*−/−*^ mice have increased BMD and bone mineral content (BMC) due to increased osteoblastic bone formation and mineral apposition rates [24]. However, despite the inverse relationship between PYY and bone density in humans and the high bone mass phenotype observed in *Y1R*^*−/−*^ mice, deletion of PYY in mice has resulted in contrasting skeletal outcomes [25,26]. In one study, genetic targeting of *Pyy* resulted in a null allele that retained a *lacZ* reporter gene and also functionally deleted pancreatic polypeptide. These mice were analysed at P60 (males), P186 (males), P210 and P275 (males and females) and displayed decreased BMD and bone volume [25]. In a second study, gene targeting resulted in retention of Cre recombinase and an EGFP reporter gene in the knockout allele. By contrast, these mice were analysed at P98 (males and females) had increased BMD and bone formation [26]. Overall, these contradictory reports have led to confusion regarding the role of PYY in the regulation of bone mass [25,26].

It is essential to clarify the role of PYY in the regulation of bone mass and strength because the use of PYY analogues in the treatment of obesity has the potential to cause long-term detrimental side-effects in the skeleton, including an increased risk of fracture. We thus determined the skeletal consequences of PYY deletion in *Pyy KO* mice, in which gene targeting resulted in a null allele lacking the entire PYY coding region but retaining only single *frt* and *loxP* sites following recombination. These mice were studied during skeletal development at postnatal day 14 and during adulthood at postnatal days 70 and 186 in both sexes.

## 2 Materials and methods

### 2.1 Animals

Global deletion of the *Pyy* gene in mice was performed using the Cre-LoxP method as described previously [13]. The resultant global *Pyy KO* mice were maintained on a C57BL/6 background. Mice were housed in a specific pathogen free facility at 22 ± 2 °C with a 12-h light/dark cycle, and ad libitum access to water and rodent chow. *Pyy KO* mice and WT littermate controls were collected at postnatal days P14, P70, P112 and P186, using humane schedule 1 methods. Tissue was collected immediately and placed in either 70% ethanol or 10% neutral buffered formalin for 24 h prior to storage in 70% ethanol. All studies were performed in accordance to the U.K. Animal (Scientific Procedures) Act 1986, the ARRIVE guidelines and the EU Directive 2010/63/EU for animal experiments.

### 2.2 Histology – Alcian blue and van Gieson staining of growth plates

Tibias were fixed for 24 h in 10% neutral buffered formalin, decalcified in 10% EDTA, and decalcification confirmed by X-ray microradiography. Samples (*n* = 5 per genotype at P14 and *n* = 4 per sex, per genotype at P70) were embedded in paraffin and 5 μm sections stained with Alcian blue and van Gieson [27,28]. Sections were imaged using a Leica DM LB2 microscope, and measurements taken from at two different levels and a minimum of four positions across the growth plate using ImageJ to determine mean heights of the growth plate zones [25].

### 2.3 Osteoclast histomorphometry

Tartrate-resistant acid phosphatase (TRAP) activity was detected in sections from decalcified paraffin embedded tibias counterstained with aniline blue (*n* = 2–3 per sex, per genotype at P70) and osteoclast parameters were determined from two separate levels using a Leica DM LB2 microscope, as described [29].

### 2.4 CD31 immunohistochemistry

Platelet endothelial cell adhesion molecule (PECAM-1, CD31) immunohistochemistry was performed using sections from decalcified paraffin embedded tibias (*n* = 4 male *Pyy-KO* mice at P70). PECAM-1 antigen retrieval was performed in citrate buffer pH 6 at 60 °C for 1 h. The primary rabbit anti-CD31 antibody ab124432 (Abcam; Cambridge UK) was diluted 1:500 in. The secondary goat anti-rabbit IgG (horseradish peroxidase) antibody ab205718 (Abcam) was diluted 1:1000. Sections were counterstained with haematoxylin and imaged using a Leica DM LB2 microscope.

### 2.5 Digital X-ray microradiography

Femurs and caudal vertebrae 6 and 7 were imaged using an MX20 Faxitron at 26 kV, 10 μm resolution (*n* = 7 per genotype at P14 and *n* = 7–8 per sex, per genotype at P70 and P186). Steel, aluminium and polyester standards were used to determine relative bone mineral content using ImageJ [27,30]. Bone lengths were determined from X-ray images using ImageJ (*n* = 7 per genotype at P14 and n = 7–8 per sex, per genotype at P70 and P186).

### 2.6 Micro-computerised tomography (microCT)

Femur trabecular bone was imaged in 70% ethanol using a Skyscan 1172a micro-computerised tomography scanner (Bruker MicroCT, Kontich, Belgium) (*n* = 5 per sex, per genotype at P112). Scans were performed at 50 kV, 200 μA, 0.5 mm aluminium filter with a detection voxel size of 4 μm^3^, and images were reconstructed using Skyscan NRecon software. Trabecular number (Tb.N), thickness (Tb.Th), and bone volume as a proportion of tissue volume (BV/TV) were calculated within a 1-mm^3^ region of interest located 0.2 mm below the growth plate [31]. Femur cortical bone was imaged in 70% ethanol using a Scanco uCT50 (Scanco Medical, Zurich, Switzerland) (*n* = 4–6 per sex, per genotype at P112). Scans were performed at 70 kV, 200 μA, 0.5 mm aluminium filter at a 10 μm voxel resolution, and images were reconstructed using Scanco software. Cortical thickness (Ct.Th), marrow cavity diameter (Ma.Dm) and cortical bone mineral density (BMD) were calculated within a 1.5 mm region of interest centered in the mid-shaft region 56% along the length of the femur distal to the femoral head. Cortical porosity scans were performed at 1 μm voxel resolution in 0.5 mm^3^ regions of interest at the mid and distal femur in male mice at P70 (*n* = 6 per sex, per genotype).

### 2.7 Back scattered electron scanning electron microscopy (BSE-SEM)

Femurs were fixed in 70% ethanol and then opened longitudinally using a plain cylinder tungsten carbide burr in a dental hand-piece and macerated in alkaline bacterial pronase at 55 °C [32]. Samples were carbon-coated and imaged using back-scattered electrons with a Zeiss DSM962 digital scanning electron micro-scope at 20-kV beam potential (KE Electronics) (*n* = 4–5 per sex, per genotype at P70). ImageJ was used to quantify osteoclastic resorption surfaces as a fraction of total endosteal and trabecular bone surfaces [27] and to determine endosteal vascular canal number and size.

### 2.8 Quantitative BSE-SEM (qBSE-SEM)

Humeri were embedded in polymethyl methacrylate (PMMA), polished to an optically flat finish, carbon coated and imaged by SEM (Tescan UK, Cambridge, UK) at high vacuum with a 4-quadrant back-scattered electron detector (Deben, UK) (*n* = 5 per sex, per genotype at P70 and P186). Images were captured at 20 kV, 0.5 nA at a working distance of 20 mm. Graduations of micro-mineralisation densities were determined by comparison to high and low molecular weight standards and visualised using an 8-interval pseudo-color scheme [27,32,33].

### 2.9 Iodine contrast enhanced BSE-SEM (ICE-BSE-SEM)

Humeri embedded in polymethyl methacrylate (PMMA), were polished to an optically flat finish, and stained with potassium iodide. Stock Lugol's Iodine solution (Pro-Lab Diagnostics) was diluted with an equal volume of absolute ethanol and pipetted directly onto the block surface. After 15 min, blocks were washed with distilled water and air dried. Blocks were imaged by SEM (Tescan UK, Cambridge, UK) at high vacuum with a 4-quadrant back-scattered electron detector (Deben, UK) (*n* = 4 per sex, per genotype at P70). Images were captured at 20 kV, 0.6 nA.

### 2.10 Confocal microscopy

Mice were double calcein labelled with 15 mg/kg calcein by intra-peritoneal injections at seven and three days prior to sacrifice. A Leica SP2 reflection confocal microscope was used at 488 nm excitation to image calcein labelling in methacrylate-embedded humeri (*n* = 4–5 per sex, per genotype at P70). The rate of mineral apposition (MAR) was determined by calculating the distance separating the two calcein labels at 20 distinct locations [27,34]. Measurements were performed using ImageJ.

### 2.11 Destructive 3-point bend testing

Tibial mechanical strength parameters were determined using three-point bend testing (*n* = 7–8 per sex, per genotype at P70 and P186). Destructive 3-point bend tests were performed on an Instron 5543 materials testing load frame using custom built mounts with rounded supports that minimize cutting and shear loads [27]. Bones were positioned horizontally and centered on the custom supports with the anterior surface upward. Load was applied vertically to the mid-shaft with a constant rate of displacement of 0.03 mm/s until fracture. A span of 12 mm was used for all adult tibia and load displacement curves were used to calculate biomechanical variables [[27], [35]].

### 2.12 Statistical analysis

Students' *t*-tests were used to determine the statistical significance of differences between experimental groups. A *P* value <0.05 was considered significant. Unless stated otherwise, data presented represents the mean value ± standard error. A Kolmogorov-Smirnov test was used to determine differences between frequency distributions of mineralisation densities from qBSE-SEM and digital X-ray microradiography images and endosteal vessel size from BSE-SEM images [27,28,30].

## 3 Results

### 3.1 Linear growth is accelerated but bone mineral content reduced in juvenile Pyy KO mice

**Fig. 1:**
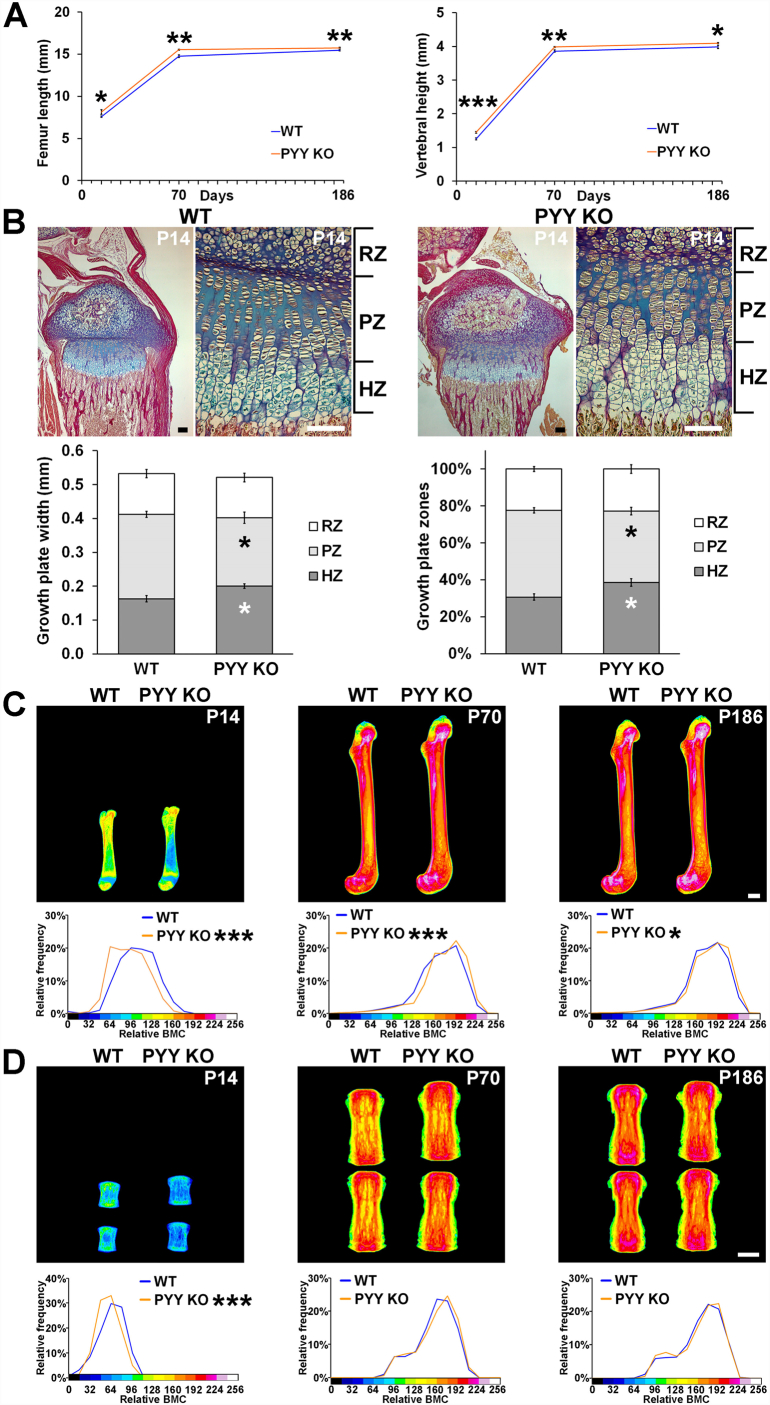
Linear growth and endochondral ossification. (A) Femur lengths and caudal vertebral heights from male and female P14, and male P70 and P186 WT and *Pyy KO* mice (mean ± SEM, *n* = 7–8 per genotype per age, *P < 0.05, **P < 0.01, ***P < 0.001 versus WT; unpaired *t*-test). (B) Decalcified sections of P14 proximal tibia stained with Alcian blue (cartilage) and van Gieson (bone matrix); scale bars = 100 μm. Graphs represent the absolute and relative widths of the resting zone (RZ), proliferating zone (PZ) and hypertrophic zone (HZ) chondrocytes in the growth plate of WT and *Pyy KO* mice (mean ± SEM, *n* = 5 per genotype, *P < 0.05, versus WT; unpaired *t*-test). (C) Representative quantitative X-ray microradiographic images of femurs from male and female P14 and male P70 and P186 WT and *Pyy KO* mice; scale bar = 1 mm. Pseudo-coloured images represent grey scale images using a 16-color interval scheme with low mineral content blue and high mineral content pink. Graphs are relative frequency histograms of bone mineral content (BMC) (*n* = 7–8 per genotype per age, *P < 0.05, ***P < 0.001 versus WT; Kolmogorov-Smirnov test). (D) Quantitative X-ray microradiographic images of vertebrae from male and female P14 and male P70 and P186 mice; scale bar = 1 mm. Relative frequency histograms of BMC (n = 7–8 per genotype per age, ***P < 0.001 versus WT; Kolmogorov-Smirnov test).

X-ray microradiography analysis demonstrated that male and female *Pyy KO* mice had increased femur length and vertebral height at P14, P70 and P183 (Fig. 1A, Supplementary Fig. 1A). Growth plate morphology was also abnormal at P14 and characterised by decreased proliferative zone (PZ) and increased hypertrophic zone (HZ) widths (Fig. 1B). Nevertheless, growth plate parameters had normalised by P70 (data not shown). Despite increased post-natal linear growth, femurs and vertebrae from *Pyy KO* mice had markedly reduced BMC at P14 (Fig. 1C and D).

### 3.2 Bone mineral content is increased in adult Pyy KO mice

In contrast to the reduced BMC in juvenile *Pyy KO* mice femurs from adult P*yy KO* mice had increased BMC (Fig. 1C and Supplementary Fig. 1B). Nevertheless, vertebral BMC was normal in adult P*yy KO* mice (Fig. 1D, Supplementary Fig. 1C).

### 3.3 Bone mass is increased in adult male Pyy KO mice

**Fig. 2:**
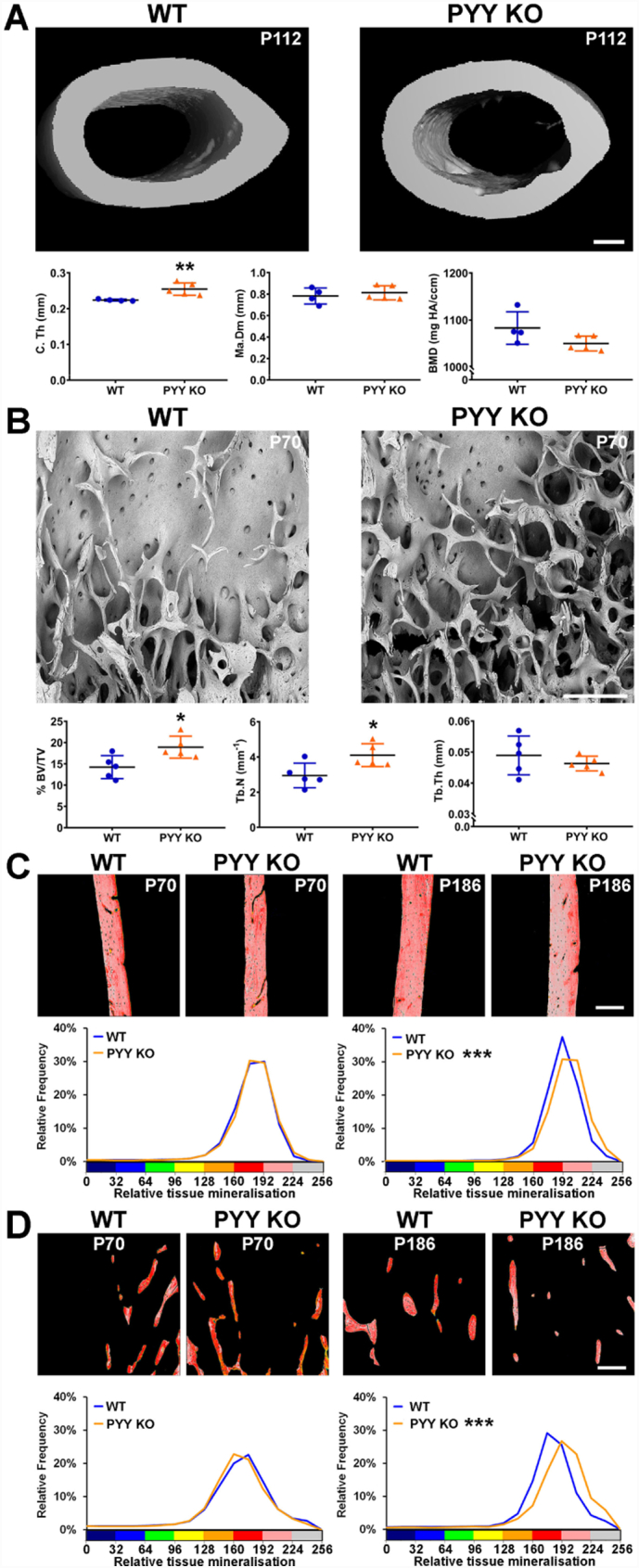
Bone structure and mineralisation. (A) Transverse micro-CT rendered images of midshaft femur from male P112 WT and *Pyy KO* mice; scale bar 200 μm. Graphs show cortical bone structural parameters; cortical thickness (C.Th), marrow cavity diameter (Ma.Dm) and bone mineral density (BMD) (mean ± SEM, *n* = 4–5 per genotype, **P < 0.01 versus WT; unpaired *t*-test). (B) Representative BSE-SEM images of distal femur trabecular bone from male P70 WT and *Pyy KO* mice (n = 4 per genotype); scale bar = 200 μm. Graphs show trabecular bone structural parameters determined by microCT; bone volume as a percentage of tissue volume (BV/TV), trabecular number (Tb.N) and trabecular thickness (Tb.Th) in male P112 WT and *Pyy KO* mice (mean ± SEM, *n* = 5 per genotype, *P < 0.05 versus WT; unpaired *t*-test). (C) Quantitative BSE-SEM images of cortical bone from the proximal humeri of male P70 and P186 WT and *Pyy KO* mice; scale bar = 200 μm. Pseudo-coloured images represent grey scale images using an 8-color interval scheme with low mineral content green/yellow and high mineral content pink/grey. Graphs are relative frequency histograms of bone micro-mineralisation densities (images representative of *n* = 5 per genotype, ***P < 0.001 versus WT; Kolmogorov-Smirnov test). (D) Quantitative BSE-SEM images of trabecular bone from the proximal humeri of male P70 and P186 WT and *Pyy KO* mice; scale bar = 200 μm. Relative frequency histograms of bone micro-mineralisation densities (images representative of n = 5 per genotype, ***P < 0.001 versus WT; Kolmogorov-Smirnov test).

At P112 micro-CT analysis demonstrated adult male *Pyy KO* mice had increased cortical and trabecular bone mass characterised by increased cortical thickness (C.Th), trabecular bone volume (BV/TV) and trabecular number (Tb.N) (Fig. 2 A and B). Nevertheless, the cortical BMD at P112 did not differ from WT (*P* = 0.096) suggesting that the increase in BMC in early adulthood is a consequence of increased cortical thickness rather than abnormal mineralisation. By contrast, at P112 cortical and trabecular bone parameters in female *Pyy KO* mice were unaffected (Supplementary Fig. 2A and B).

qBSE-SEM analysis demonstrated normal cortical and trabecular bone mineralisation in male and female *Pyy KO* at the time of cessation of linear growth (P70), whereas mineralisation was elevated at both skeletal sites in males (Fig. 2C and D) and females at P186 (Supplementary Fig. 2C and D).

### 3.4 Bone formation is increased in Pyy KO mice

**Fig. 3:**
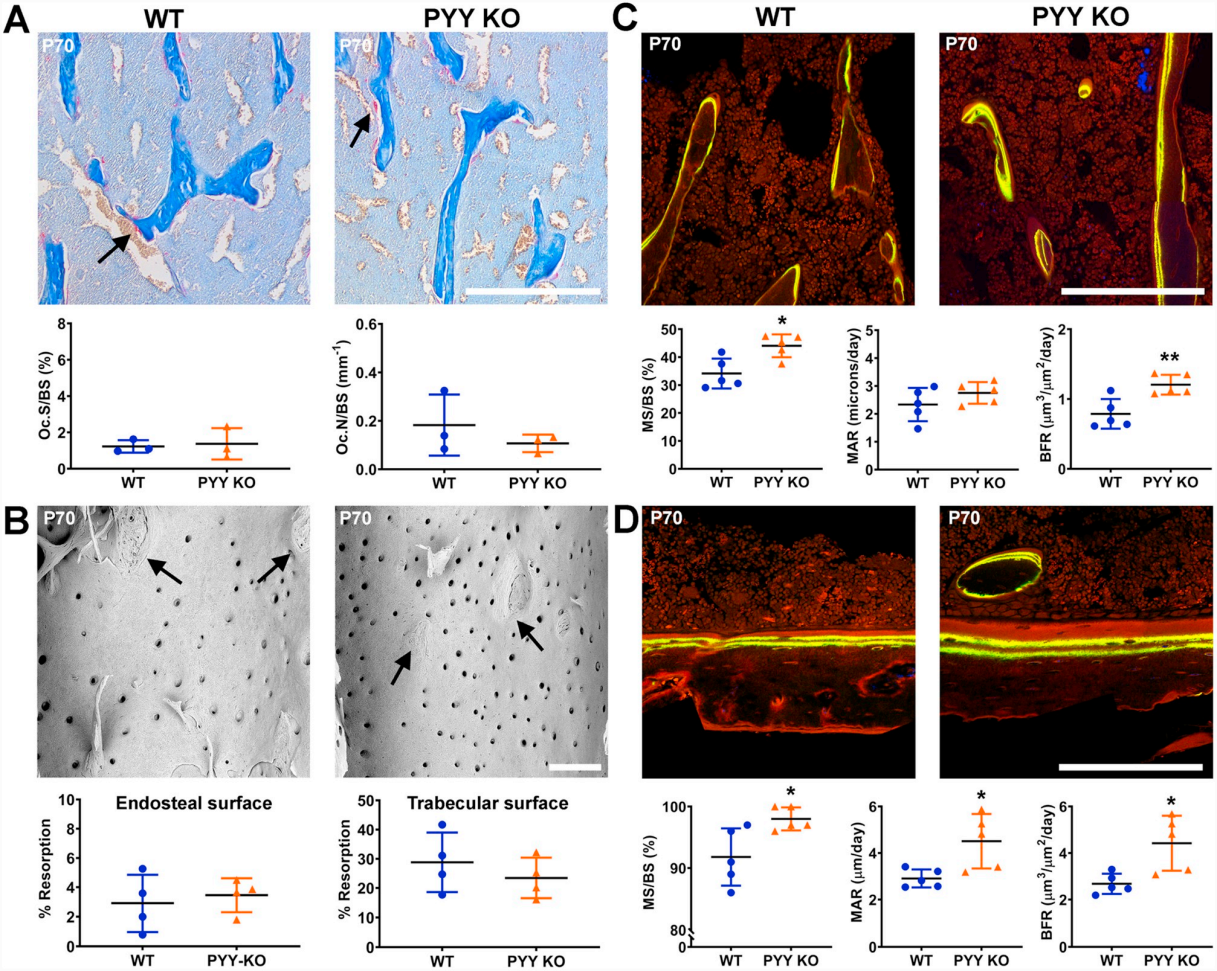
Osteoclastic bone resorption and osteoblastic bone formation. (A) Decalcified sections of P70 proximal tibia from male P70 WT and *Pyy KO* mice stained for tartrate-resistant acid phosphatase (TRAP); scale bar = 200 μm. Black arrow indicates examples of red TRAP-stained osteoclasts. Graphs show numbers of osteoclasts per mm bone surface (OcN/BS) and osteoclast surface per mm bone surface (OcS/BS) in male WT and *Pyy KO* mice (mean ± SEM, *n* = 3 per genotype). (B) BSE-SEM images of femur endosteal bone surfaces from male P70 WT and *Pyy KO* mice. Arrows indicate borders between regions of osteoclastic resorption and unresorbed bone surfaces; scale bar = 200 μm. Graphs show endosteal and trabecular resorption surfaces as percentage of total endosteal and trabecular bone surface respectively (mean ± SEM, *n* = 4 per genotype). (C) Confocal images of trabecular bone from proximal humerus of male P70 WT and *Pyy KO* mice double-labelled with calcein; scale bar = 200 μm. Graphs show trabecular mineralising surface (MS/BS), mineral apposition rate (MAR) and bone formation rate (BFR) (mean ± SEM, *n* = 5 per genotype, *P < 0.05, **P < 0.01 versus WT; unpaired *t*-test). (D) Confocal images of cortical bone from proximal humerus of male mice; scale bar = 200 μm. Graphs show cortical MS/BS, MAR and BFR (mean ± SEM, n = 5 per genotype, *P < 0.05, versus WT; unpaired *t*-test).

To investigate the cellular mechanism underlying the increased bone mass and mineralisation observed in adult *Pyy KO* mice, we performed static and dynamic histomorphometry at P70. TRAP staining revealed no differences in osteoclast number or surface in either male or female *Pyy KO* mice compared to WT (Fig. 3A, Supplementary Fig. 3A). Furthermore, osteoclast endosteal and trabecular resorption surfaces in male and female *Pyy KO* mice were similar to WT at P70 (Fig. 3B, Supplementary Fig. 3B) and P186 (Data not shown). By contrast, confocal analysis of calcein double labelling demonstrated increased mineralising surfaces (MS/BS) and bone formation rates (BFR) in trabecular bone and increased MS/BS mineral apposition rate (MAR) and BFR in cortical bone from male P70 *Pyy KO* mice (Fig. 3C and D). Bone formation parameters in female P70 *Pyy KO* mice were normal (Supplementary Fig. 3C and D).

### 3.5 Increased bone strength and cortical vascularity in Pyy KO mice

**Fig. 4:**
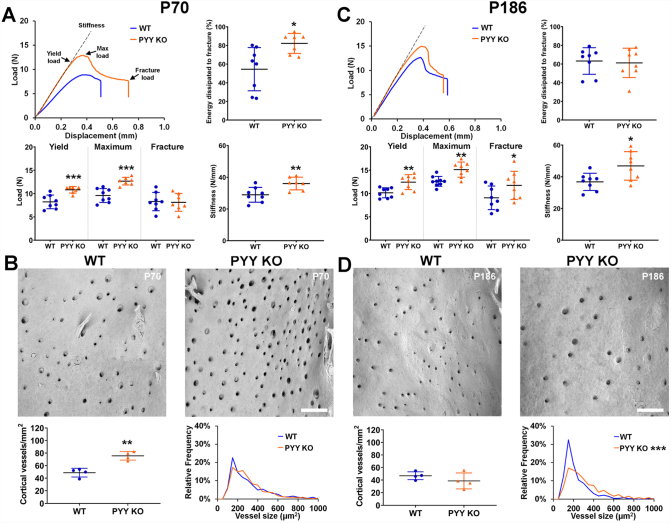
Bone strength and cortical vascularity. (A) Representative load displacement curves from 3-point bend testing of tibias from male P70 WT and *Pyy KO* mice. Graphs show yield, maximum and fracture loads, stiffness and energy dissipated prior to fracture (toughness) (mean ± SEM, *n* = 7–8 per genotype, *P < 0.05, **P < 0.01, ***P < 0.001 versus WT; unpaired *t*-test). (B) BSE-SEM images of mid-femur endosteal surfaces from male P70 WT and *Pyy KO* male mice; scale bar = 200 μm. Graphs show endosteal surface vessel density and relative frequency histogram of vessel size (mean ± SEM, *n* = 4 per genotype, **P < 0.01 versus WT; unpaired *t*-test). (C) Representative load displacement curves from 3-point bend testing of tibias from male P186 WT and *Pyy KO* mice. Graphs show yield, maximum and fracture loads, stiffness and energy dissipated prior to fracture (toughness) (mean ± SEM, n = 7–8 per genotype, *P < 0.05, **P < 0.01, versus WT; unpaired *t*-test). (D) BSE-SEM images of mid-femur endosteal surfaces from female P186 WT and *Pyy KO* mice; scale bar = 200 μm. Graphs show endosteal surface vessel density and relative frequency histogram of vessel size (mean ± SEM, n = 4 per genotype, ***P < 0.001 versus WT; Kolmogorov-Smirnov test).

Biomechanical testing revealed increased bone strength and toughness characterised by increased yield and maximum loads, stiffness and energy dissipated prior to fracture (toughness) in both male and female P70 *Pyy KO* mice compared to WT (Fig. 4A, Supplementary Fig. 4A). In addition to the increase in bone strength and toughness at P70 in *Pyy KO* mice, cortical bone vascularity was also increased by 56% in males and by 64% in females (Fig. 4B, Supplementary Fig. 4B). Nevertheless, the size of these cortical vascular channels did not differ from WT. Biomechanical testing in older animals demonstrated a persistent increase in bone strength with higher yield, maximum and fracture loads and stiffness in male *Pyy* KO mice (Fig. 4C) and higher maximum and fracture loads in female *Pyy* KO mice (Supplementary Fig. 4C). In male P186 *Pyy* KO mice the number of cortical vascular canals was similar to WT mice but they were larger in size (Fig. 4D) whereas, in female P186 *Pyy* KO mice both the number and size of cortical vascular channels was increased (Supplementary Fig. 4D).

### 3.6 Increased cortical porosity in Pyy KO mice

**Fig. 5:**
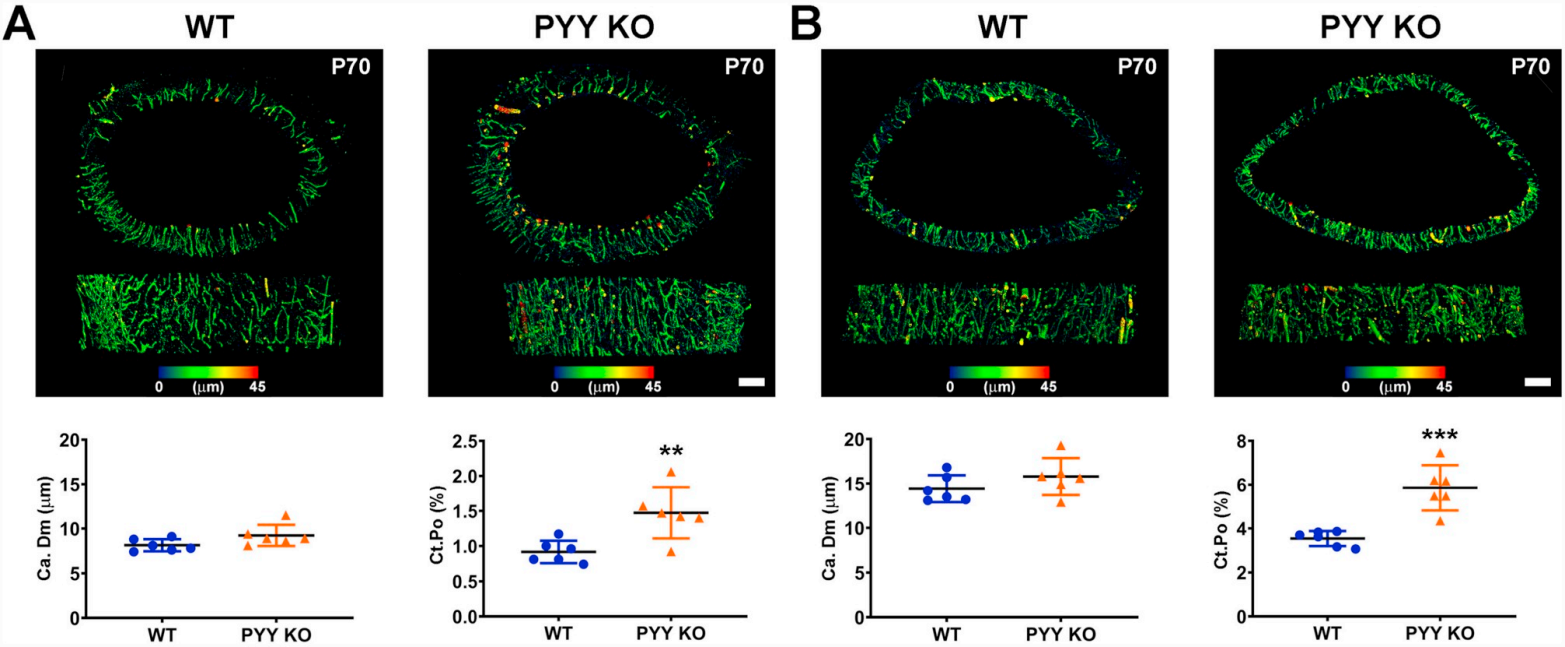
Cortical porosity. (A) Transverse and anterior-posterior micro-CT images showing cortical bone porosity in the mid-femoral diaphysis of male P70 *Pyy KO* and WT mice; bars = 200 μm. Graphs show mean canal diameter (Ca.Dm) and cortical porosity (Ct.Po) (Pore volume/Cortical bone volume (Po·V/Ct.V)) (mean ± SEM, *n* = 6 per genotype, **P < 0.01 versus WT; unpaired *t*-test). (B) Micro-CT images showing cortical bone porosity in distal-femoral diaphysis of male P70 *Pyy KO* and WT mice; bars = 200 μm. Graphs show mean canal diameter (Ca.Dm) and cortical porosity (Ct.Po) (mean ± SEM, n = 6 per genotype, ***P < 0.001 versus WT; unpaired *t*-test).

To further investigate the abnormal cortical bone vascularity, we determined cortical porosity in the mid and distal femur diaphysis in male P70 *Pyy* KO mice. Although the mean canal diameter (Ca.Dm) did not differ between *Pyy* KO and WT mice, porosity was increased by 61% in both the mid and distal femur diaphysis in *Pyy KO* mice compared to WT (Fig. 5A and B). Importantly, there was no evidence of cortical trabecularisation of the endosteal surface in male or female mice (Fig. 3B and 4B and D, Supplementary Fig. 3B and 4 B and D). We used CD31 (PECAM-1) immunohistochemistry and high resolution iodine contrast enhanced BSE-SEM to demonstrate that the greater cortical porosity observed in *Pyy KO* mice resulted, at least in part, from increased vascularity (Supplementary Fig. S5).

## 4 Discussion

PYY is a gut hormone secreted from gastrointestinal enteroendocrine L-cells in response to nutrient intake [10]. The PYY(3–36) isoform inhibits appetite and food intake and is currently being evaluated for the treatment of obesity [36]. Importantly, the inverse relationship between circulating PYY and BMD raises the possibility of detrimental long-term side-effects affecting bone mass and strength in individuals treated with PYY analogues [21,22]. Here we show that adult *Pyy KO* mice have increased bone mass, mineralisation and strength despite increased cortical bone porosity, demonstrating that PYY is a negative regulator of adult bone maintenance.

### 4.1 Clarification of the role of PYY in bone

Previous studies investigating the role of PYY in bone have been contradictory. Wortley et al. [25] reported decreased bone mineral content and density in young adult and mature male *Pyy KO* mice at P60 and P186, and in both male and female *Pyy KO* mice at P210 whereas Wong et al. [26] demonstrated increased bone volume, mineral apposition rate and bone formation rate in adult male and female *Pyy KO* mice at P98. The current study clarifies this confusion, extends the findings reported by Wong et al., and is consistent with decreased bone mass in patients with increased circulating PYY [21,22,26].

The reason for the contrasting phenotypes described in previous studies [25,26] is unclear, although the different ages of the animals analysed was raised as a potential confounding factor. To address this by we analysed mice during growth (P14), at cessation of growth (P70) and during adulthood (P112 and P186). An alternative explanation for the divergent skeletal phenotypes observed in previous studies may be the use of different gene targeting approaches used to delete *Pyy*. Wortley et al. [25] replaced the *Pyy* coding region with a *lacZ* reporter gene that is retained in the knockout allele, with mice maintained on a C57BL/6 background. This approach also resulted in functional deletion of pancreatic polypeptide. By contrast, Wong et al. [26] generated knockout mice that retained the Cre recombinase gene and an EGFP reporter in the knockout allele [37]. Here we used *Pyy KO* mice with deletion of the entire coding region but without retention of a selection marker or expression cassette. These mice were backcrossed and maintained on a pure C57BL/6 background [13]. Thus the current study definitively identifies PYY as a negative regulator of bone mass, mineralisation and strength.

Moreover, the contrasting gene targeting approaches result in contrasting secondary metabolic effects, which may also contribute to the divergent skeletal phenotypes. Wortley et al. [25] reported no change in food intake, body composition or serum insulin level when *Pyy KO* mice were maintained on a standard chow diet. Wong et al. [26], however, reported sexual dimorphism with increased body weight, reduced food intake and increased serum insulin restricted to females [37]. The *Pyy KO* mice used in the current study have previously been shown to exhibit increased food intake, body weight and leptin levels from 5 weeks of age [13]. The potential reasons for the metabolic differences between three strains of *Pyy KO* mice have been discussed in depth previously [13]. Nevertheless, the similar skeletal abnormalities reported here and by Wong et al. [26] suggest they are a direct consequence of deficient PYY signalling in the skeleton rather than secondary to a systemic metabolic phenotype. Consistent with this, we show that the underlying cellular mechanism for the increased bone mass in *Pyy KO* mice is enhanced osteoblastic bone formation and mineral apposition. Accordingly, increased mineralisation has been reported previously in primary osteoblast cultures obtained from *Y2*^*−/−*^ mice [38] and enhanced ERK signalling has been shown in primary WT osteoblasts treated with PYY [26,39].

### 4.2 PYY is a negative regulator of linear growth

Here we show that bone length is increased in juvenile and adult *Pyy KO* mice, findings that are consistent with the decreased bone length previously reported in PYY over-expressing *PYYtg* mice [26]. These data suggest a novel role for PYY in regulation of endochondral ossification and linear growth that is supported by the increased hypertrophic differentiation in the growth plates of *Pyy KO* mice. Of note and in contrast to the increased bone mass in adult *Pyy KO* mice, juveniles had decreased bone mass indicating bone mineral accrual is impaired during this period of abnormal linear growth.

### 4.3 PYY regulates cortical bone vascularity

Increased cortical bone porosity is frequently associated with high bone turnover osteoporosis and fragility fracture and has been reported in patients with anorexia nervosa, who have elevated PYY levels [40,41]. We unexpectedly also demonstrated increased cortical porosity in adult *Pyy KO* mice but found it was due to increased vascularity. In contrast to the cortical porosity, cortical trabecularisation and fragility seen in high bone turnover osteoporosis, the increased vascularity in *Pyy KO* mice at P70 was accompanied by normal bone resorption, increased bone formation, and increased cortical thickness with normal mineralisation. These increases in bone strength and stiffness at P70 were unexpectedly associated with a marked increase in the energy dissipated prior to fracture, suggesting that the increased toughness may result from the abnormal cortical bone porosity. Nevertheless, at P186 when cortical mineralisation was increased in *Pyy KO* mice, toughness did not differ from WT animals indicating that bone strength and toughness is determined by a complex interplay between cortical diameter, thickness, mineralisation and porosity. Previous studies have shown PYY mediates vasoconstriction via Y1 or Y2 receptor [42] consistent with the increased cortical vessel size in P186 *Pyy KO* mice*.* Furthermore, the increase in cortical vessel number in P70 *Pyy KO* mice suggests PYY may also inhibit angiogenesis during bone growth.

In conclusion, these data demonstrate PYY is a negative regulator of adult bone mass and strength and suggest that BMD should be monitored in patients following bariatric surgery or in individuals receiving PYY analogue treatment.

## Appendix A Supplementary data

Supplementary figures Image 1
