# Supplementary material for: PYY is a negative regulator of bone mass and strength
Source: Bone. 2019 Oct;127:427–35. doi: 10.1016/j.bone.2019.07.011 (PMC6715792; doi:10.1016/j.bone.2019.07.011)
Supplement: Supplementary file 1 — Supplementary figures [file mmc1.docx]

**Supplementary material**

**
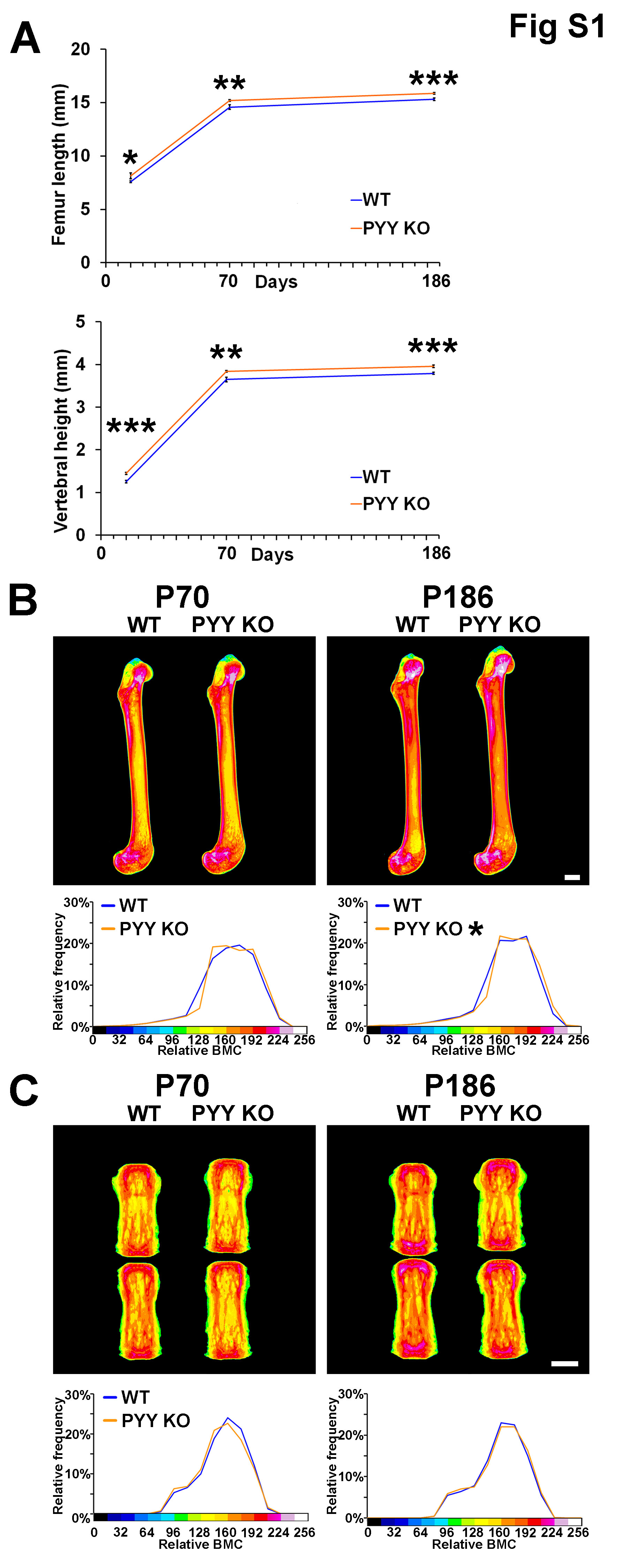
Supplementary Figure 1: Linear growth and endochondral ossification in female PYY KO mice**

**(A)** Femur lengths and caudal vertebral heights from male and female P14, and female P70 and P186 WT and *Pyy KO* mice (mean ± SEM, n=7–8 per genotype per age, **P*<0.05, ***P*<0.01, ****P*<0.001 versus WT; unpaired *t*-test).

**(B)** Quantitative X-ray microradiographic images of femurs from female P70 and P186 WT and *Pyy KO* mice; scale bar = 1 mm. Pseudo-coloured images represent grey scale images using a 16-color interval scheme with low mineral content blue and high mineral content pink. Graphs are relative frequency histograms of bone mineral content (BMC) (n=7–8 per genotype per age, **P*<0.05 versus WT; Kolmogorov-Smirnov test).

**(C)** Quantitative X-ray microradiographic images of vertebrae from female P70 and P186 mice; scale bar = 1 mm. Relative frequency histograms of BMC (n=7–8 per genotype per age).

**
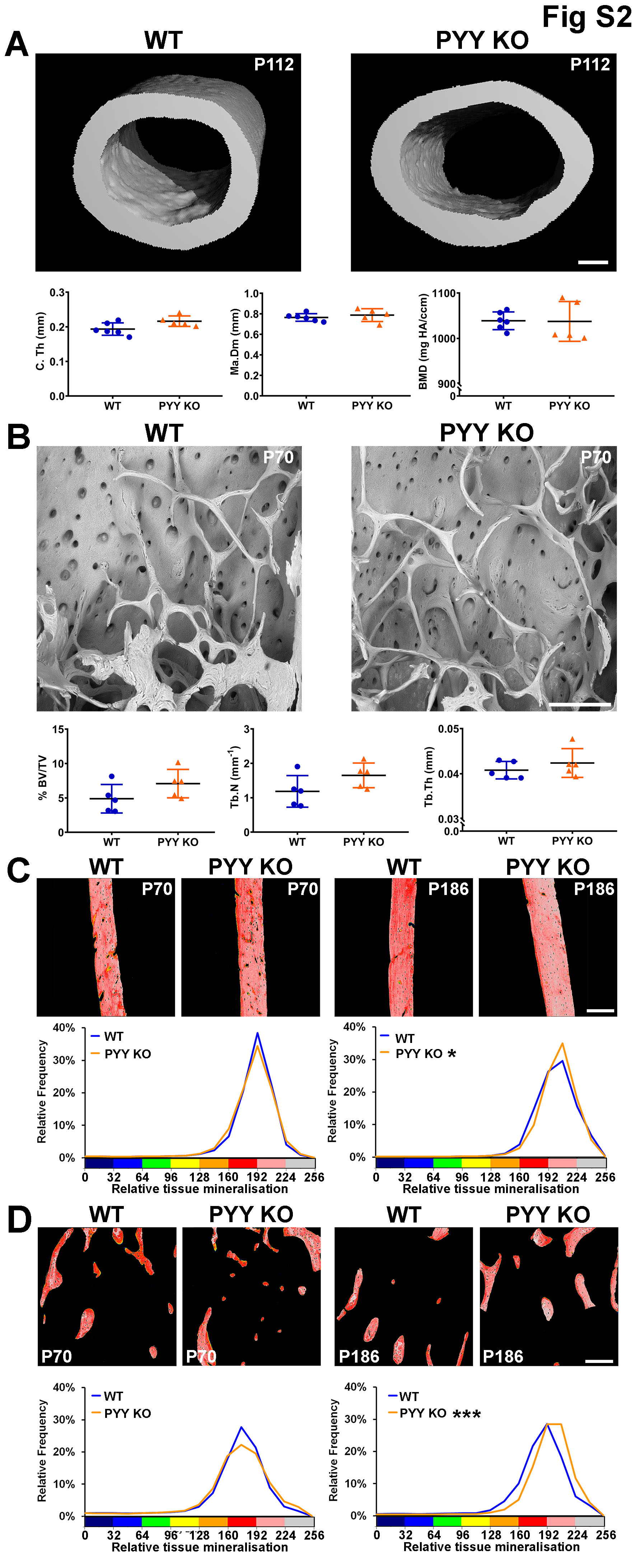
Supplementary Figure 2: Bone structure and mineralisation in female PYY-KO mice**

**(A)** Transverse Micro-CT rendered images of midshaft femur female in P112 WT and *Pyy KO* mice; scale bar 200 μm. Graphs show cortical bone structural parameters; cortical thickness (C.Th), marrow cavity diameter (Ma.Dm) and bone mineral density (BMD) (mean ± SEM, n=6 per genotype).

**(B)** Representative BSE-SEM images of distal femur trabecular bone from female P70 WT and *Pyy KO* mice (n=5 per genotype); scale bar = 200 μm. Graphs show trabecular bone structural parameters determined by microCT; bone volume as a percentage of tissue volume (BV/TV), trabecular number (Tb.N) and trabecular thickness (Tb.Th) in female P112 WT and *Pyy KO* mice (mean ± SEM, n=5 per genotype).

**(C)** Quantitative BSE-SEM images of cortical bone from the proximal humerus of female P70 and P186 WT and *Pyy KO* mice; scale bar = 200μm. Pseudo-coloured images represent grey scale images using an 8-color interval scheme with low mineral content green/yellow and high mineral content pink/grey. Graphs are relative frequency histograms of bone micro-mineralisation densities (images representative of n=5 per genotype, **P*<0.05 versus WT; Kolmogorov-Smirnov test).

**(D)** Quantitative BSE-SEM images of trabecular bone from the proximal humerus of female P70 and P186 WT and *Pyy KO* mice; scale bar = 200μm. Graphs are relative frequency histograms of bone micro-mineralisation densities (images representative of n=5 per genotype, ****P*<0.001 versus WT; Kolmogorov-Smirnov test)

**
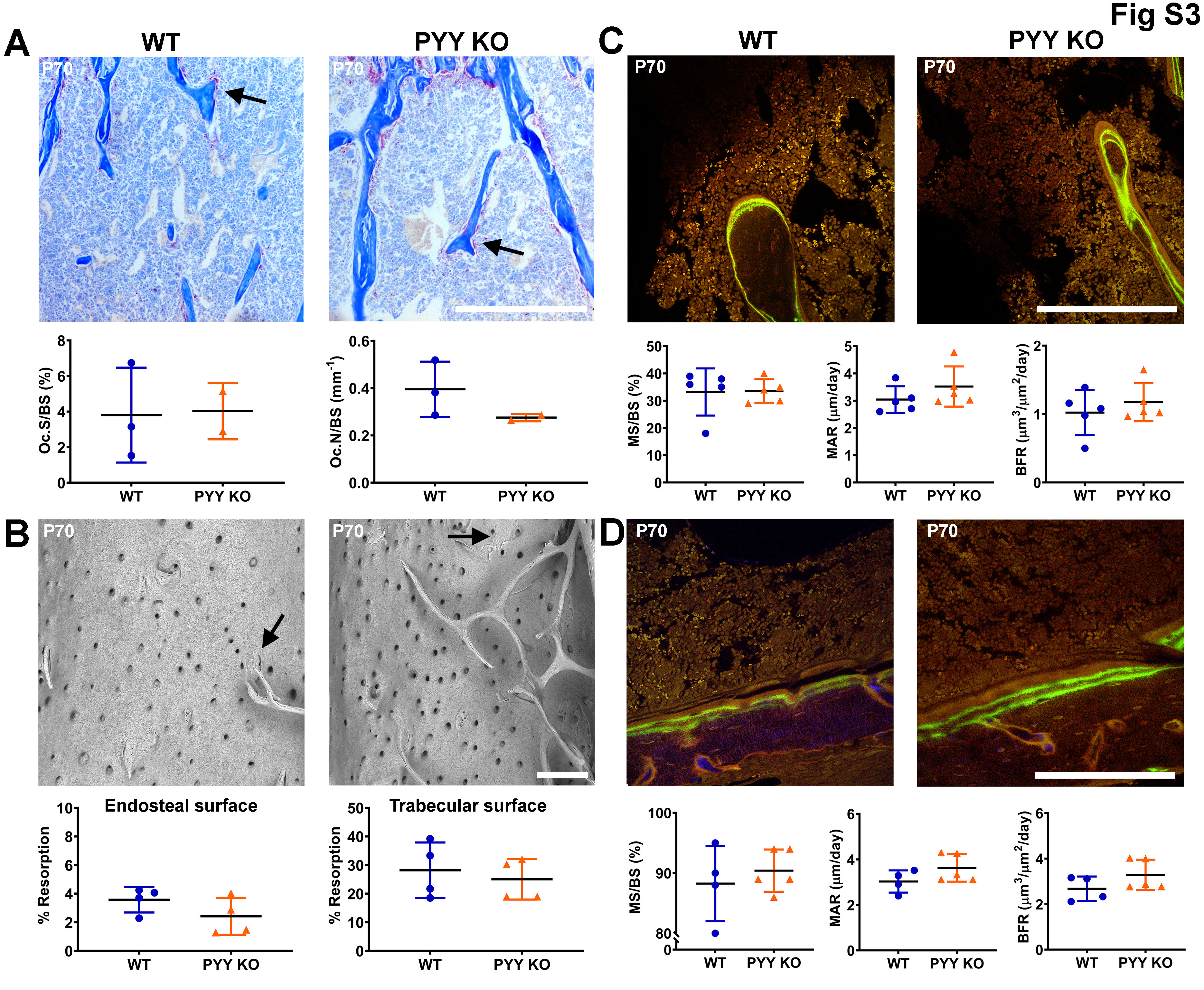
**

**Supplementary Figure 3: Osteoclastic bone resorption and osteoblastic bone formation in female PYY-KO mice**

**(A)** Decalcified sections of P70 proximal tibia from female P70 WT and *Pyy KO* mice stained for tartrate-resistant acid phosphatase (TRAP); scale bar = 200 μm. Black arrow indicates examples of red TRAP-stained osteoclasts. Graphs show numbers of osteoclasts per mm bone surface (OcN/BS) and osteoclast surface per mm bone surface (OcS/BS) in male WT and *Pyy KO* mice (mean ± SEM, n=2-3 per genotype)

**(B)** BSE-SEM images of femur endosteal bone surfaces from female P70 WT and *Pyy KO* mice. Arrows indicate borders between regions of osteoclastic resorption and unresorbed bone surfaces; scale bar = 200 μm. Graphs show endosteal and trabecular resorption surfaces as percentage of total endosteal and trabecular bone surface respectively (mean ± SEM, n=4 per genotype).

**(C)** Confocal images of trabecular bone from proximal humerus of female P70 WT and *Pyy KO* mice double-labelled with calcein; scale bar = 200μm. Graphs show trabecular mineralising surface (MS/BS), mineral apposition rate (MAR) and bone formation rate (BFR) (mean ± SEM, n=5 per genotype).

**(D)** Confocal images of cortical bone from proximal humerus in female mice; scale bar = 200μm. Graphs show cortical MS/BS, MAR and BFR (mean ± SEM, n=4-5 per genotype).

**
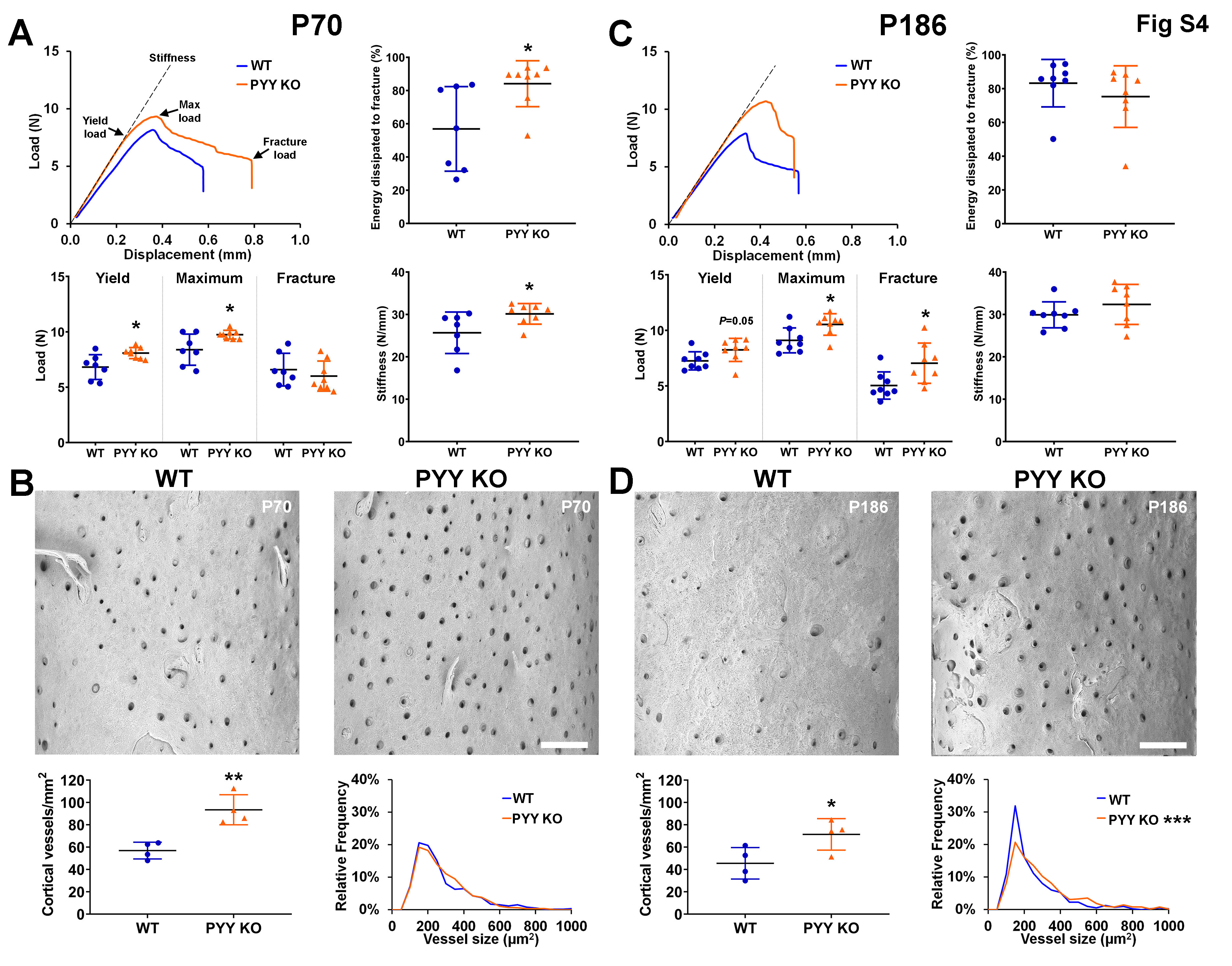
**

**Supplementary Figure 4: Bone strength in female PYY KO mice**

**(A)** Representative load displacement curves from 3-point bend testing of tibias from female P70 WT and *Pyy KO* mice. Graphs show yield, maximum and fracture loads, stiffness and energy dissipated prior to fracture (toughness) (mean ± SEM, n=7–8 per genotype, **P*<0.05, versus WT; unpaired *t*-test).

**(B)** BSE-SEM images of mid-femur endosteal surfaces from female P70 WT and *Pyy KO* male mice; scale bar = 200 μm. Graphs show endosteal surface vessel density and relative frequency histogram of vessel size (mean ± SEM, n=4 per genotype, ***P*<0.01 versus WT; unpaired *t*-test).

**(C)** Representative load displacement curves from 3-point bend testing of tibias from female P186 WT and *Pyy KO* mice. Graphs show yield, maximum and fracture loads, stiffness and energy dissipated prior to fracture (toughness) (mean ± SEM, n=7–8 per genotype, **P*<0.05, versus WT; unpaired t-test).

**(D)** BSE-SEM images of mid-femur endosteal surfaces from female P186 WT and *Pyy KO* mice; scale bar = 200 μm. Graphs show endosteal surface vessel density and relative frequency histogram of vessel size (mean ± SEM, n=4 per genotype, **P*<0.05 versus WT; unpaired t-test and ****P*<0.001 versus WT; Kolmogorov-Smirnov test)

**
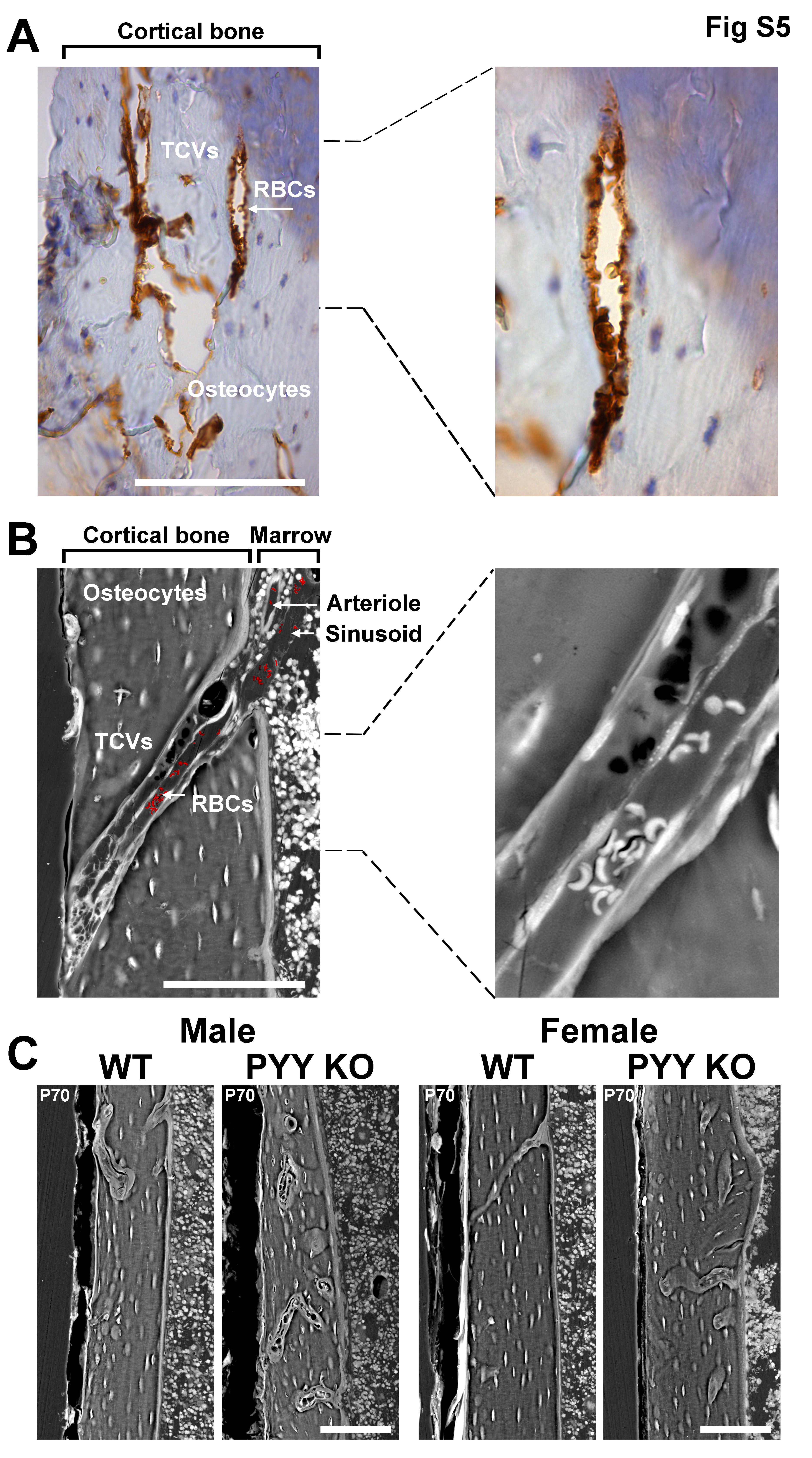
**

**Supplementary Figure 5: Increased trans-cortical vessels in *PYY KO* mice**

**(A)** Left panel shows decalcified sections of P70 proximal tibia from male P70 *Pyy KO* mice stained for CD31 (PECAM-1) showing trans-cortical vessels; scale bar = 100 μm. Right panel shows higher magnification of the TCV and intraluminal RBCs

**(B)** Left panel shows iodine contrast enhanced BSE-SEM image showing trans-cortical vessels (TCVs) in humerus cortical bone from a P70 WT mouse. Red blood cells (RBCs) have been pseudocoloured red; scale bar = 100 μm. Right panel shows higher magnification of the TCV and intraluminal RBCs.

**(C)** Representative iodine contrast enhanced BSE-SEM images showing trans-cortical vessels (TCVs) in humerus cortical bone from male and female P70 *Pyy KO* and WT mice; scale bar = 100 μm.
